# Interactions between the invasive Burmese python, *Python bivittatus* Kuhl, and the local mosquito community in Florida, USA

**DOI:** 10.1371/journal.pone.0190633

**Published:** 2018-01-17

**Authors:** Lawrence E. Reeves, Kenneth L. Krysko, Michael L. Avery, Jennifer L. Gillett-Kaufman, Akito Y. Kawahara, C. Roxanne Connelly, Phillip E. Kaufman

**Affiliations:** 1 Entomology and Nematology Department, Institute of Food and Agricultural Sciences, University of Florida, Gainesville, Florida, United States of America; 2 Division of Herpetology, Florida Museum of Natural History, University of Florida, Gainesville, Florida, United States of America; 3 National Wildlife Research Center, United States Department of Agriculture, Gainesville, Florida, United States of America; 4 McGuire Center for Lepidoptera and Biodiversity, Florida Museum of Natural History, University of Florida, Gainesville, Florida, United States of America; 5 Florida Medical Entomology Laboratory, Institute of Food and Agricultural Sciences, University of Florida, Vero Beach, Florida, United States of America; Institut Pasteur, FRANCE

## Abstract

The Burmese python, *Python bivittatus* Kuhl, is a well-established invasive species in the greater Everglades ecosystem of southern Florida, USA. Most research on its ecological impacts focuses on its role as a predator and its trophic interactions with native vertebrate species, particularly mammals. Beyond predation, there is little known about the ecological interactions between *P*. *bivittatus* and native faunal communities. It is likely that established populations of *P*. *bivittatus* in southern Florida serve as hosts for native mosquito communities. To test this concept, we used mitochondrial cytochrome c oxidase subunit I DNA barcoding to determine the hosts of blood fed mosquitoes collected at a research facility in northern Florida where captive *P*. *bivittatus* and Argentine black and white tegu, *Salvator merianae* (Duméril and Bibron), are maintained in outdoor enclosures, accessible to local mosquitoes. We recovered python DNA from the blood meals of three species of *Culex* mosquitoes: *Culex erraticus* (Dyar and Knab), *Culex quinquefasciatus* Say, and *Culex pilosus* (Dyar and Knab). *Culex erraticus* conclusively (P = 0.001; Fisher’s Exact Test) took more blood meals from *P*. *bivittatus* than from any other available host. While the majority of mosquito blood meals in our sample were derived from *P*. *bivittatus*, only one was derived from *S*. *merianae*. These results demonstrate that local mosquitoes will feed on invasive *P*. *bivittatus*, a recently introduced host. If these interactions also occur in southern Florida, *P*. *bivittatus* may be involved in the transmission networks of mosquito-vectored pathogens. Our results also illustrate the potential of detecting the presence of *P*. *bivittatus* in the field through screening mosquito blood meals for their DNA.

## Introduction

Introductions of plant and animal species to areas outside their native ranges can destabilize ecosystems [1, 2], contribute to biotic homogenization [3], impose public health risks [4], and impose large economic costs through direct damage or mitigation efforts [5]. Introduced animal species interact with various ecological components within ecosystems. Direct competitive and trophic interactions are traditionally considered the primary drivers of invasive species-associated impacts. However, direct or indirect interactions with native pathogens or parasites similarly may lead to damaging impacts and may go undetected. Species introductions have the potential to dramatically alter the prevalence or transmission systems of parasites and pathogens through the introduction of novel vectors [6], spillover of co-introduced pathogens from introduced hosts to native hosts [7], or spillback of endemic pathogens to native hosts through competent introduced reservoir hosts [8].

In the United States, Florida is particularly susceptible to species introductions [9, 10]. The state has both tropical and subtropical climates, major international ports of entry for tourists and commercial goods from the Caribbean, South America and elsewhere, active horticultural and captive wildlife industries, and empty niches created by human altered environments [11]. These factors facilitate the introduction and establishment of many non-native species, particularly, amphibian and reptile taxa. Currently, at least 180 non-native herpetofaunal taxa have been introduced to the state and 63 are established, more than in any other U.S. state or global region [12]. Some of these species, such as the Burmese python, *Python bivittatus*, and Argentine black and white tegu, *Salvator merianae*, are high profile invasive species that have attracted public attention and scientific interest [13].

*Python bivittatus* is presumed to have been introduced to the Flamingo area of Everglades National Park prior to 1985 and became established thereafter [14–16]. *Python bivittatus* is an opportunistic predator of mammals, birds and reptiles. In Florida, its diet consists of both small and large animals including protected and managed species, such as wading birds, alligators and deer, and federally protected species [17–20]. Research on the ecological impacts of *P*. *bivittatus* are largely limited to its trophic interactions and direct effects on prey populations. The establishment and expansion of python populations is correlated with precipitous declines in relative abundance of several mammal species [21–23]. Through the diminished abundance or extirpation of mammalian mesopredators, a cascade of python-mediated indirect effects is possible [24].

**Fig 1:**
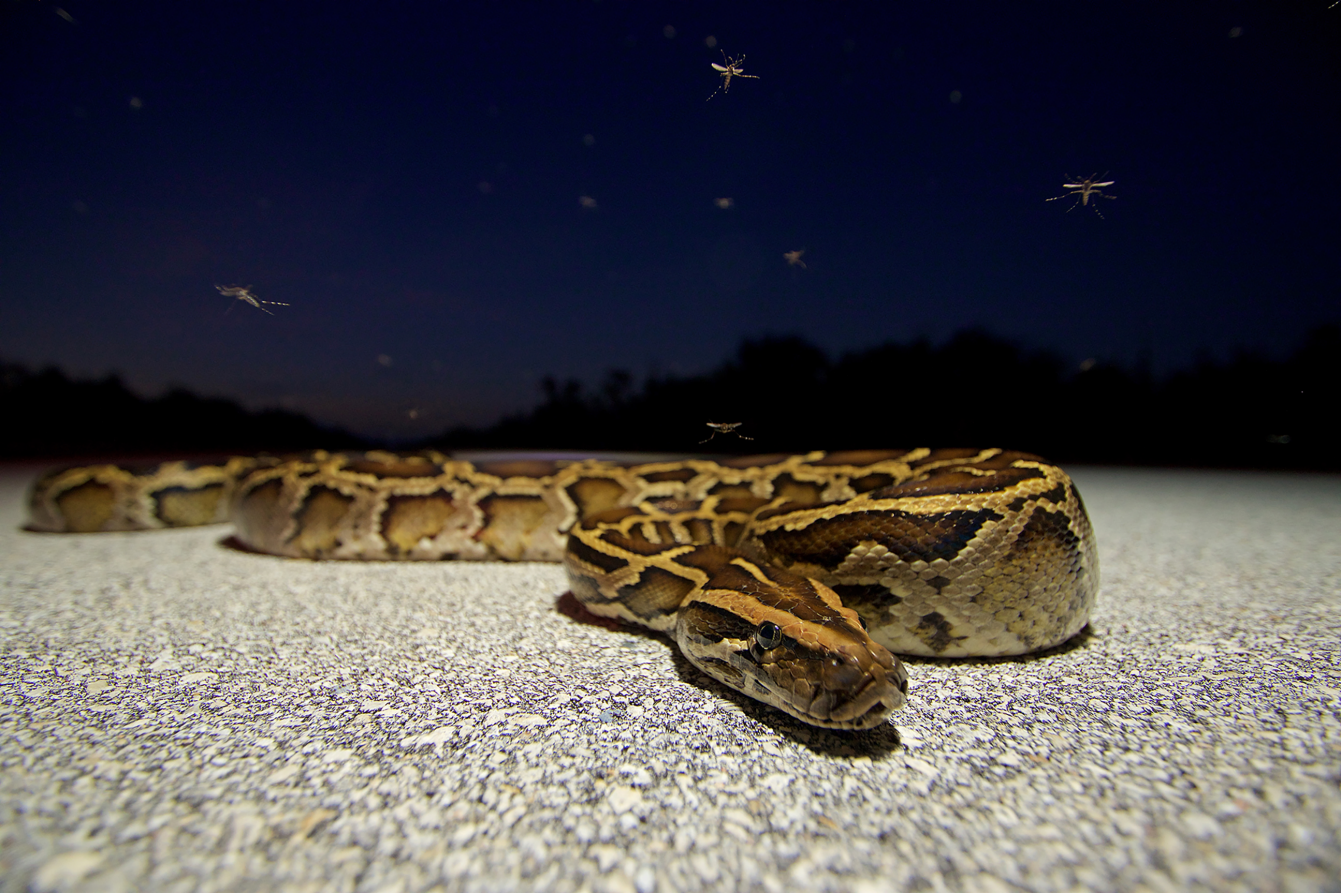
Burmese python (*Python bivittatus*) and *Aedes taeniorhynchus* mosquitoes in Everglades National Park, October 2015. In the Greater Everglades Ecosystem of southern Florida, introduced populations of *P*. *bivittatus* are sympatric with a local mosquito fauna that includes more than 50 native and introduced mosquito species.

The integration of a novel, large-bodied predator into the ecosystems of southern Florida has the potential to alter the transmission dynamics of endemic parasites and pathogens. In Florida, mosquitoes are the vectors of numerous zoonotic parasites and pathogens, many of which are poorly known. Species diversity and composition of vertebrate host communities are among the factors that structure transmission networks and affect the prevalence of mosquito-vectored pathogens [25]. In much of southern Florida, *P*. *bivittatus* is an available host for a diverse mosquito fauna (Fig 1) that includes more than 50 native and introduced mosquito species, some of which are potential pathogen vectors [26]. Females of many North American mosquito species take blood meals from reptilian hosts, and some specialize entirely on ectothermic hosts [27, 28]. Through mosquito feeding, there is potential that pythons are exposed to mosquito-vectored parasites and pathogens and it is possible that *P*. *bivittatus* is now directly involved in the transmission of endemic parasites and pathogens. Further, by dramatically restructuring the vertebrate host community and eliminating some host taxa through predation, the establishment of *P*. *bivittatus* may have broader implications for mosquito-borne pathogen transmission in Florida.

The diversity, host range, vectors, and transmission dynamics of many mosquito-vectored reptile pathogens are poorly known. While no known data are available on the pathogens of *P*. *bivittatus* in its native or introduced range, squamates (lizards and snakes) in Florida are the hosts of many mosquito-borne parasites including species of the taxonomically diverse genera *Hepatozoon* (Apicomplexa: Adeleorina) and *Plasmodium* (Apicomplexa: Haemosporida) [29–31]. Similarly, the role of snakes in the transmission systems of medically-important mosquito-borne viruses is not well understood. However, snakes are competent hosts for the medically-important zoonoses Eastern equine encephalitis virus, Western equine encephalitis virus and West Nile virus, and may promote the persistence of these viruses within an ecosystem [32–35].

The intent of this study is to determine if local mosquito communities interact with *P*. *bivittatus*, an introduced host. Native mosquito species have been documented feeding from other exotic reptile species in Florida [36], but interactions between *P*. *bivittatus* and mosquitoes are not known in the state. Determining whether native mosquitoes feed upon *P*. *bivittatus* and identifying those species is the first step towards understanding any potential pathogen transmission implications of python establishment in Florida. Using traditional survey methods (i.e., transect walks, road surveys), *P*. *bivittatus* is difficult to detect in the habitats it occupies. Environmental DNA approaches using water sampling have been proposed to improve the ability of detecting *P*. *bivittatus* at field sites in southern Florida [37, 38]. If mosquitoes take blood meals from *P*. *bivittatus*, mosquito blood meals could be used as an alternative source of environmental DNA in monitoring python populations.

## Materials and methods

### Mosquito sampling

**Fig 2:**
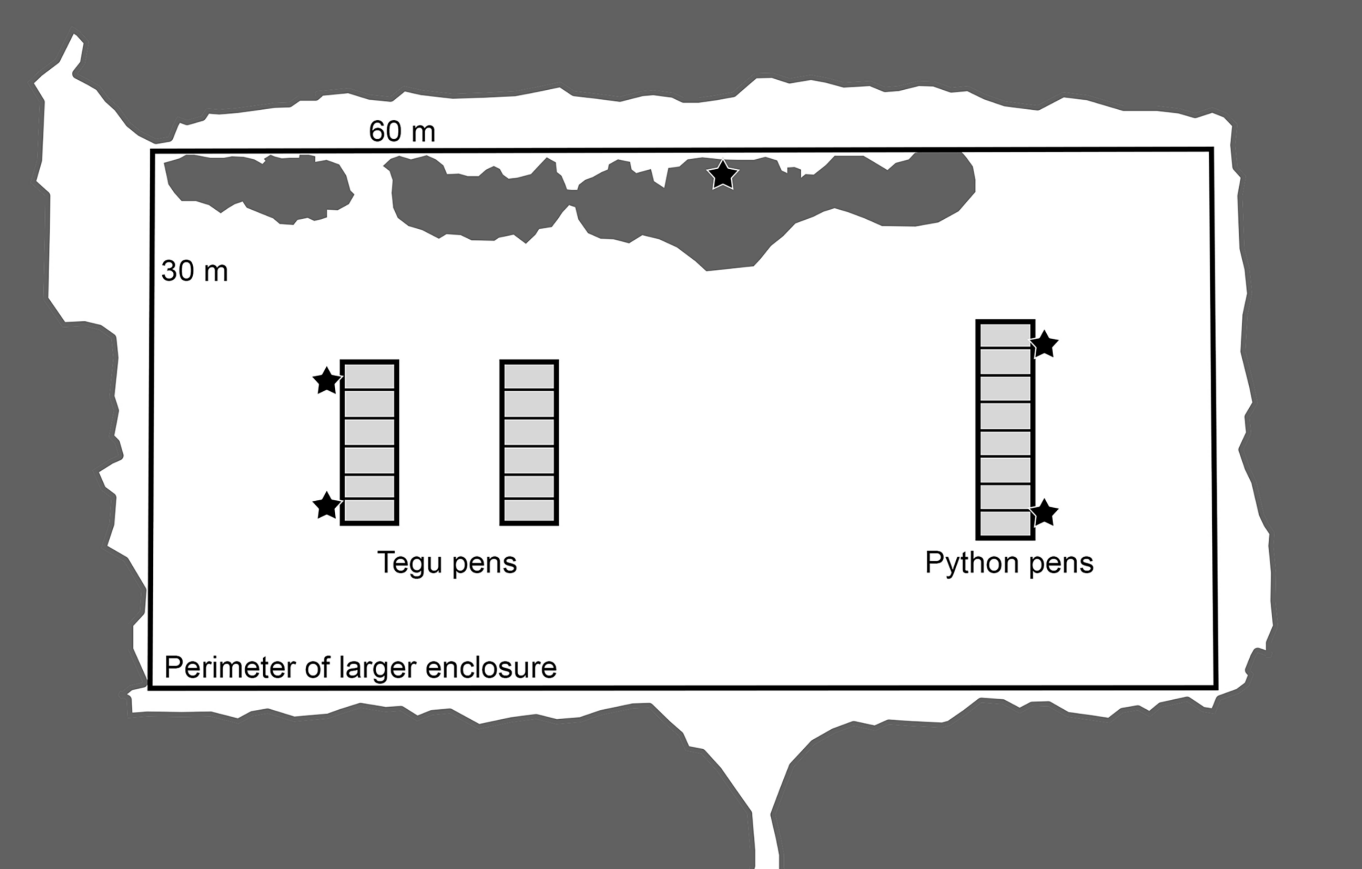
Location of mosquito resting shelter traps in relation to reptile pens. Arrangement (from above) of pens (light gray shaded boxes) housing Burmese (*Python bivittatus*) or Argentine black and white tegu (*Salvator merianae*) where blood fed mosquitoes were collected in resting shelter traps (stars) at the USDA National Wildlife Research Center in Gainesville, Florida, USA. Pens were set within the perimeter of a 60 × 30 × 4 m aviary-like enclosure (dark outer line). Two shelter traps were placed alongside python pens, two alongside tegu pens, and one in a saw palmetto thicket between the two pens. Areas that are shaded darker gray indicate areas of natural, unmaintained vegetation. Unshaded areas indicate mowed grassy areas.

Mosquitoes were collected at the United States Department of Agriculture (USDA), National Wildlife Research Center (NWRC) (29° 39' 14.6", N 82° 17' 17.1" W; datum WGS84, elev. 45 m), in Gainesville, Alachua County, Florida, USA. At this facility, eight adult *P*. *bivittatus* and 12 adult *S*. *merianae* were housed individually in outdoor pens arranged in three parallel rows (Fig 2) where they were accessible to the local mosquito community. One row of eight adjacent python pens (1.5 m wide × 3.0 m deep × 1.8 m high) was located approximately 20 m from two rows of six adjacent tegu pens (1.5 m wide × 3.0 m deep × 1.8 m high). Rows of tegu pens were approximately 4 m apart. Pen walls and ceilings consisted of polyvinyl chloride (PVC) coated steel wire mesh with square openings 2.54 × 2.54 cm. Each reptile pen included an animal shelter. Python pens each contained one 1 × 1 × 0.5 m insulated plastic box with a 15 × 30 cm opening. Tegu pens each had one artificial burrow consisting of a 1.5 m tunnel made from 10.2 cm diameter corrugated plastic tubing leading to an underground chamber (48 × 66 × 33 cm). All python and tegu pens were positioned within a larger, aviary-like enclosure (60 × 30 × 4 m) of fiber netting (mesh size ~2.5 cm). Vegetation within this enclosure and surrounding reptile pens was limited to grasses consistently maintained by mowing, a few small thickets of saw palmetto, *Serenoa repens*, and young hardwood trees. The vegetation immediately surrounding the larger enclosure consisted of a pine flatwoods community with a canopy of *Pinus palustris*, and *Pinus elliotti*, interspersed with hardwoods, and an understory dominated by saw palmetto.

Mosquitoes were collected from July-October 2015 and April-December 2016. Mosquitoes were not collected from November 2015–March 2016 when snakes were relocated to indoor enclosures to avoid potentially cold temperatures. Five resting shelter traps were placed inside the larger enclosure. Two traps were located alongside the python pens, two alongside the tegu pens, and one in a saw palmetto thicket (Fig 2). Resting shelter traps were constructed after Burkett-Cadena [39], and consisted of a 1 × 2 m section of 5.1 × 10.2 cm steel fencing, rolled into a cylindrical frame, set inside a 159 L black heavy-duty trash bag to make a cylinder, open at one end. The trash bag was secured tightly to the frame with duct tape. To remove mosquitoes from the traps, the open end of the resting shelter was covered by a 76 × 102 cm piece of cardboard that had a 9 cm circular hole cut in the center. The hole was positioned over the opening of the trap and a battery-powered aspirator, made from a modified 12V vacuum (BioQuip Products, Rancho Domiguez, CA), was turned on and held parallel to the hole, immediately outside the trap. The trap was then shaken vigorously to flush resting mosquitoes. The aspirator collected mosquitoes attempting to escape the shelter through the hole in the cardboard cover. After shaking, the cardboard cover was removed and the aspirator, still running, was moved to the interior of trap to collect any mosquitoes that had not attempted to exit through the hole in the cover.

We collected mosquitoes from one to three times per week, between 0800 and 0900 and immediately transported them 10.8 km to the Entomology and Nematology Department, University of Florida, Gainesville, Florida, USA. Collected mosquitoes were killed within 30 minutes of collection by exposure to ethyl acetate for approximately 10 minutes. Mosquitoes were visually inspected under a stereoscope for the presence of a blood meal and identified to species using a morphological taxonomic key to adult females [26]. Gut contents of blood fed mosquitoes were preserved on Whatman^®^ four-sample Flinders Technology Associates (FTA) cards [40]. Each blood fed mosquito was assigned a unique number and transferred to the sampling area of an FTA card. For blood fed females, a sterile pipette tip was used to apply pressure to the abdomen, releasing the blood meal onto the card. The blood meal was spread over the sampling area of the FTA card with the pipette tip until all viscous droplets were absorbed. The extent of blood meal digestion was visually estimated by scoring blood meals on a rank of 1–3; an approach modified from Detinova [41]. The following scores were based on the stage of digestion after feeding: (1) fresh blood meal, bright red in color, large size in proportion to mosquito abdomen and absence of visible developing ovaries; (2) blood meal that was still red in color, but developing ovaries were apparent; and (3) smaller blood meal, characterized by a brownish or blackish color, and ovaries that occupied more than 50% of the abdomen.

A subset of the collected male and unfed female mosquitoes (n = 23, representing all species collected) were processed identically and served as negative controls, with the contents of the abdomen spread over the sampling area of the FTA card with a sterile pipette tip. FTA cards were then labeled and stored at room temperature until DNA extraction.

### Blood meal analysis

DNA was extracted following the HotSHOT method [42] using hot sodium hydroxide to lyse cells and a Tris-hydrogen chloride neutralization buffer solution. For each FTA card-preserved blood meal, a hole punch was used to remove two circular 1 mm diameter sections of the FTA card sampling area. The two FTA-card sections were transferred to a 0.2 mL tube with flame-sterilized forceps. Initially, 50 μL of lysis solution (25 mM NaOH and 0.2 mM EDTA) was added to each tube. Tubes were then incubated in a BioRad^®^ DNA Engine thermocycler at 95°C for 30 minutes followed by 4°C for five minutes. Thereafter, 50 μL of neutralization solution (40 mM Tris-HCl) was added to each tube and vortexed for ~10 s. Extracted DNA was stored at -20°C until PCR.

A degenerate primer set [43] was used to amplify a 664 base pair (bp) fragment of the vertebrate host cytochrome *c* oxidase subunit I gene (COI). Three nucleotide mismatches at the forward priming site of mosquito templates inhibited co-amplification of mosquito DNA. The primers RepCOI-F (5’-TNT TMT CAA CNA ACC ACA AAG A-3’) and RepCOI-R (5’-ACT TCT GGR TGK CCA AAR AAT CA-3’) were used in 20 μL reactions each consisting of 4 μL 10X PCR buffer, 0.6 μL 50 mM MgCl_2_, 0.4 μL of 10 mM dNTPs, 0.48 μL of each 10 mM primer, 0.2 μL of Taq polymerase (5 U/μL), 1 μL of extracted DNA, and 12.84 μL of sterile, double distilled water. Reaction conditions followed a standard profile of 94°C for 3 min, followed by 40 cycles of 94°C for 40 s, 48.5°C for 30 s, and 72°C for 60 s, and a final extension step of 72°C for 7 min. Reaction products were stained with ethidium bromide and visualized under ultra-violet light on a 1.5% agarose gel. A 50 bp DNA ladder (Invitrogen^TM^) was used to determine the approximate fragment size of PCR products. Negative controls in which sterile, double-distilled water was used in place of extracted DNA were included in all reactions to monitor for contamination. All PCR products a visible band at the expected fragment size were sent to the University of Florida Interdisciplinary Center for Biotechnology Research, Gainesville, Florida, for Sanger sequencing on an ABI 3130^®^ automated sequencer. Resulting sequencing chromatograms were examined for quality and edited to truncate ambiguous terminal stretches from the sequence using the software Geneious^®^ Version R10 [44]. Edited sequences were searched on the National Center for Biotechnology Information (NCBI) GenBank database (https://www.ncbi.nlm.nih.gov/genbank/) using the Basic Local Alignment Search Tool (BLAST). Species-level taxonomic identities were assigned to blood meals when a host sequence was ≥ 98% homologous to a sequence referenced in the database [45] or sequences obtained from museum specimens.

Statistical analyses were performed in the statistical program R^®^ Version 3.2.0 using the stats package [46]. For each mosquito species that was determined to feed from *P*. *bivittatus*, we used Fisher’s Exact Test to compare the proportion of blood meals derived from *P*. *bivittatus* to the proportion derived from all other identified host species. Results of these tests were considered significant if p < 0.05. No permits were required for the described study, which complied with all relevant regulations.

## Results

We collected a total of 511 adult female mosquitoes using resting shelter traps (325 traps nights) from the USDA NWRC. This sample represented seven species in four genera (Table 1). *Culex erraticus* was the most abundant species collected and represented 72% of all adult females collected. *Culex quinquefasciatus*, and to a lesser extent, *Cx*. *pilosus* and *Aedes albopictus* also were common, together representing 24% of the total sample. *Aedes infirmatus*, *Anopheles crucians*, and *Uranotaenia lowii* were collected in small numbers (< 10 individuals, each species). We observed other mosquito species (including *Mansonia* sp., *Coquillettidia perturbans*, *Ps*. *ciliata*, *Psorophora ferox*, and an *Aedes* (*Ochlerotatus*) species) searching for hosts or feeding from humans at the site, but these species were absent from our resting shelter samples.

**Table 1:** Taxonomic composition of female mosquitoes collected in resting shelters at the USDA National Wildlife Research Center, Gainesville, Florida, USA over 325 trap nights between July 2015 and December 2016.

Mosquito Species	# Collected	Mean per day (S.D.)	# Blood fed (%)	Mean # blood fed per day (S.D.)
*Aedes albopictus*	23	0.35 (0.65)	0 (0)	-
*Aedes infirmatus*	9	0.14 (0.39)	0 (0)	-
*Anopheles crucians*	8	0.12 (0.38)	2 (25)	0.03 (0.17)
*Culex erraticus*	369	5.67 (2.71)	51 (14)	0.69 (1.13)
*Culex pilosus*	26	0.4 (0.66)	5 (19)	0.077 (0.27)
*Culex quinquefasciatus*	75	1.15 (1.62)	3 (4)	0.046 (0.21)
*Uranotaenia lowii*	1	0.015 (0.12)	0 (0)	-
Total	511		61	57

For each mosquito species, the total number of blood fed individuals, overall mean number of adult females collected per day, total number of blood fed individuals and overall daily mean of blood fed individuals are indicated.

Sixty-one (12.3%) of the 511 adult female mosquitoes in our sample had visible blood meals. The majority of blood-fed specimens were *Culex erraticus* (n = 51; 84% of blood fed specimens), while five or fewer (< 8% of blood fed specimens) blood-fed *An*. *crucians*, *Cx*. *pilosus*, and *Cx*. *quinquefaciatus* were collected. *Aedes albopictus*, *Ae*. *infirmatus*, and *Ur*. *lowii* were not represented in our sample of blood fed mosquitoes.

Polymerase chain reactions successfully amplified a DNA template of the expected target size (664 bp) from 57 of 61 (93.4%) mosquito blood meal samples, but failed to produce an amplicon for DNA templates from four blood meals. The likely cause of unsuccessful reactions was an advanced extent of digestion as all four blood meals received a digestion score of 3. The taxonomic origin of host DNA from all other blood meals was identified through Sanger sequencing. Edited chromatograms of host sequences had distinct and well-defined peaks with little background noise, indicating that the sequences were of good quality. No evidence of multiple host feedings was detected in the sequencing chromatograms. The majority of sequences and all sequences derived from *P*. *bivittatus*, were 99–100% similar to referenced sequences in the NCBI database. Ten vertebrate host species representing the classes Reptilia, Aves, and Mammalia were identified (Table 2). *Python bivittatus* was identified as the host in 78.4% of blood meal samples. Only one blood meal was attributed to *S*. *merianae*. Twelve blood meals had been acquired from other hosts, including humans. Three mosquito species (*Cx*. *erraticus*, *Cx*. *pilosus*, *Cx*. *quinquefasciatus*) had obtained blood meals from *P*. *bivittatus*, while no python blood meal was detected in either *An*. *crucians* specimen sampled. Three blood meal sequences from *Cx*. *erraticus* and *Cx*. *pilosus* specimens were ~95% homologous to NCBI-referenced *Anolis carolinensis* sequences. To confirm that these blood meals were derived from *A*. *carolinensis*, their sequences were subsequently aligned to corresponding sequences obtained from *A*. *carolinensis* museum specimens (UF-Herpetology 170869, 170871), resulting in ≥99% homology.

**Table 2:** Host use of blood fed female mosquitoes collected at the USDA National Wildlife Research Center, Gainesville, Florida, USA between July 2015 and December 2016.

Host species	Host class	*Cx*. *erraticus*	*Cx*. *pilosus*	*Cx*. *quinquefasciatus*	*An*. *crucians*
*Anolis carolinensis*	Reptilia	1	2	0	0
*Coluber constrictor*	Reptilia	1	0	0	0
*Python bivittatus*	Reptilia	40	2	2	0
*Salvator merianae*	Reptilia	1	0	0	0
*Cathartes aura*	Aves	1	1	0	0
*Toxostoma rufum*	Aves	0	0	1	0
*Didelphis virginiana*	Mammalia	2	0	0	0
*Homo sapiens*	Mammalia	1	0	0	0
*Felis catus*	Mammalia	1	0	0	0
*Sylvilagus floridanus*	Mammalia	0	0	0	1
Unidentified		3	0	0	1
**Total**		51	5	3	2

Values represent the number of individual blood meals for each mosquito species derived from a host species.

In *Cx*. *erraticus*, the number of blood meals derived from *P*. *bivittatus* was greater than those from all other hosts combined. Forty *Cx*. *erraticus* had fed on *P*. *bivittatus*, compared to eight that fed from other hosts. At this site, where *P*. *bivittatus* was an available host, *Cx*. *erraticus* was significantly (P = 0.0002) more likely to feed on *P*. *bivittatus* than any other identified host. *Culex pilosus* and *Cx*. *quinquefasciatus* each had taken blood meals from *P*. *bivittatus*, but neither was more likely to feed on pythons over other identified host species, although the sample size for each species was small (n < 5).

## Discussion

Mosquito species vary in their host-use patterns and vectorial competency to pathogens [47]. The transmission networks of mosquito-vectored pathogens are, in large part, structured by these factors and the diversity of vertebrate communities within an ecosystem [48]. The presence of introduced vertebrate species has direct and indirect implications for mosquito-vectored pathogens [8]. An introduced species that is a competent host for a pathogen may serve as a reservoir of infection, facilitating its transmission to native hosts. Alternatively, introduced species that are not competent can serve as dead-end hosts resulting in reduced pathogen circulation. Introduced vertebrates also can have indirect effects on pathogen transmission networks if they influence or restructure host communities. In southern Florida, where there are >50 species of native and introduced mosquitoes, interactions between mosquitoes and the introduced *P*. *bivittatus* have not been previously documented. A first step towards characterizing these interactions is a determination of the mosquito species that are likely to feed on *P*. *bivittatus*.

We identified *P*. *bivittatus* DNA in the blood meals of three *Culex* species (*Cx*. *erraticus*, *Cx*. *pilosus*, and *Cx*. *quinquefasciatus*) collected at a facility in northern Florida that housed *P*. *bivittatus* in outdoor enclosures, suggesting that when pythons are available hosts, they are likely to be fed upon by local Florida mosquitoes. All three *Culex* mosquitoes are widely distributed across the southeastern coastal plain and occur throughout Florida. In southern Florida, they are sympatric with established populations of *P*. *bivittatus* [26]. *Culex* mosquitoes take blood meals from mammals, birds, reptiles and amphibians in Florida [49]. There is variation among *Culex* species in their degree of host specialization and preference for certain host classes. Some species feed from a narrow range of vertebrate hosts, while others are relative generalists. We also collected blood-fed *An*. *crucians*, but the sample size was small, and only one blood meal was identified, as *Sylvilagus floridanus*. *Anopheles crucians*, like other studied *Anopheles* mosquitoes, feeds predominantly from mammalian hosts. Only one blood meal specimen, from *Cx*. *erraticus*, contained *S*. *merianae* DNA. *Salvator merianae* is diurnal, and retreats to underground burrows at night or during unfavorable environmental conditions. Edman et al. [50] suggested that the cavity-roosting habits of woodpeckers make them less susceptible to mosquito feeding. *Culex erraticus* and *Cx*. *quinquefasciatus* search for and feed from hosts nocturnally [51]. Therefore, by spending nights underground, tegus may similarly avoid host-seeking mosquitoes. Alternatively, the artificial burrows inside the enclosures may provide suitable resting sites for mosquitoes that had fed from sleeping tegus, making them disinclined to leave the burrows before blood meals are digested.

*Culex erraticus* conclusively took more blood meals from *P*. *bivittatus* than from any other identified host. This mosquito feeds opportunistically; previous studies have reported either birds or mammals as frequent hosts, with smaller proportions of reptilian, and to a lesser extent, amphibian hosts recorded [27, 52–56]. Combined with our results, this suggests that *P*. *bivittatus* is a likely host for *Cx*. *erraticus* in southern Florida, where the species co-occur. Sample size was small for both *Cx*. *pilosus* and *Cx*. *quinquefasciatus*, but *P*. *bivittatus* DNA was recovered from two of five, and two of three blood meals, respectively. *Culex quinquefasciatus* is ornithophilic, and feeds predominantly from birds, especially passerines [49, 57], although reports of frequent mammalian, and occasionally reptilian host use exist [58]. Host associations for *Cx*. *pilosus* are not well known, but the available data suggest it feeds primarily on reptiles, particularly lizards [52].

In the southeastern U.S., *Cx*. *erraticus* and *Cx*. *quinquefasciatus* are medically-important vectors of arboviruses and parasites. *Culex erraticus* is suspected to be an important bridge vector for the Eastern equine encephalitis virus [59]. In North America, Eastern equine encephalitis virus is circulated and amplified among bird populations by the primary vector, *Culiseta melanura*, which is ornithophilic and largely specializes on passerines. The virus can escape bird-mosquito transmission to infect mammals and reptiles through competent, more generalist mosquito species. Recent evidence suggests that snakes may be important to the transmission dynamics and overwintering of Eastern equine encephalitis virus [27, 28, 35, 60] and West Nile virus [61]. The overwintering mechanisms of these viruses are unknown. Because these viruses are not transmitted directly from mosquito to mosquito, they may persist through the winter, in the absence of mosquitoes, in infected host animals that remain viremic for extended time periods, such as reptiles [34]. *Culex quinquefasciatus* is important in the transmission of a wide range of human and wildlife pathogens including viruses (West Nile virus, St. Louis encephalitis virus), avian malaria parasites, and filarial nematodes. A recent competency study suggested that *Cx*. *quinquefasciatus* may be a vector for Zika virus [62], but others have found contradictory results [63, 64].

Our results suggest that *P*. *bivittatus*, as a host for medically-important mosquito vectors, is likely to be exposed to arboviruses and other endemic pathogens and may influence pathogen transmission within its introduced range. While little is known about wildlife pathogens transmitted by mosquitoes, there are records of *Cx*. *quinquefasciatus* transmitting *Hepatozoon* blood parasites between boid snakes and lizards in Brazil [65]. In the Pantanal of South America, various mosquito species in the *Culex* subgenus *Melanoconion*, which includes *Cx*. *erraticus*, are the vectors of another reptile-specific *Hepatozoon* species, *H*. *caimani* [66]. *Culex erraticus* is also a vector of the lizard malaria parasite *Plasmodium floridense* [67]. Introduced mosquito species may affect vertebrate hosts and the transmission of local pathogens [68]. In southern Florida, >10 species of nonnative mosquitoes are present and their host-use patterns, in many cases, are not well characterized.

In Florida, *P*. *bivittatus* may indirectly influence the transmission dynamics of mosquito-vectored pathogens through predation of mammalian hosts. There is evidence that predation of mammals by *P*. *bivittatus* increases contact between the vectors and reservoir hosts of Everglades virus, a strain of the Venezuelan equine encephalitis virus [69]. Everglades virus circulates in an enzootic transmission cycle between reservoir rodent hosts via the mosquito *Culex cedecei* [70]. Reservoir hosts, specifically the cricetid rodents *Sigmodon hispidus* and *Peromyscus gossypinus*, become infected with the virus, and effectively transmit it to the *Cx*. *cedecei* mosquitoes that feed from them. This virus is currently not considered a substantial public health threat, although human populations living near native habitats that support virus circulation show high levels of exposure [71]. In humans, Everglades virus infection causes febrile symptoms that occasionally progress to neurologic disease [72]. Although *Cx*. *cedecei* feeds largely on rodents, it also feeds on other mammals including raccoons, opossums, deer, and rabbits [52]. Populations of some mammal species have declined precipitously since the introduction of the Burmese python [22, 23], correlating with a shift in the host use of *Cx*. *cedecei* towards the reservoir hosts of Everglades virus, and potentially leading to an increase in the prevalence of Everglades virus [69]. Currently, there are limited data on abundance trends of the rodent reservoir hosts of Everglades virus. We speculate that python-mediated declines in or extirpations of mammal species could affect the transmission and prevalence of Everglades virus in southern Florida by increasing or decreasing contact between vector mosquitoes and reservoir hosts. For example, predation of rodents by pythons could reduce the abundance of reservoir hosts (leading to a decrease in virus prevalence); predation of mammalian mesopredators by pythons could release reservoir rodents from mesopredator predation pressure (leading to an increase in virus prevalence); or mammal declines could push mammalophilic mosquito species that may serve as bridge-vectors, toward the rodent reservoir hosts through the decreased availability of non-reservoir mammals (leading to an increase in virus prevalence). In addition to Everglades virus, many other poorly known arboviruses (e.g., Mahogany Hammock virus, Pahayokee virus, Shark River virus, Gumbo Limbo virus) occur in mosquito-native mammal transmission cycles in the Everglades [73–75], and their transmission dynamics may be influenced by python predation of mammalian hosts.

Field surveys for *P*. *bivittatus* in difficult-to-access habitats are challenging and resource-intensive. In the Everglades, *P*. *bivittatus* is elusive, cryptic and semi-aquatic, making it difficult to detect through direct observation or traditional methods. For these reasons, molecular approaches through environmental DNA analyses have been developed that are designed to detect the presence of small fragments of python DNA in water [37, 38]. Our results demonstrate that local mosquito communities use *P*. *bivittatus* as a host. Assuming these interactions are occurring in the Everglades, mosquito blood meals could be used as an alternate source of DNA for detecting the presence of *P*. *bivittatus*. Although blood fed mosquitoes cannot be obtained as easily as water samples, one advantage of screening mosquito blood meals for python DNA is that host DNA is more concentrated and less degraded, potentially providing the ability to attribute DNA sequences to individual snakes [76], which may enable estimations of population size. Further, such an approach to *P*. *bivittatus* detection would generate datasets that are simultaneously valuable to both biodiversity monitoring and to the epidemiology of mosquito-vectored pathogens in southern Florida.

The feasibility of *P*. *bivittatus* detection through mosquito blood meals depends on the extent to which mosquitoes and pythons interact in the Greater Everglades Ecosystem of southern Florida. The habitats, mosquito and vertebrate host communities, and climatic conditions of southern Florida differ from those in northern Florida, where this research was performed. Southern Florida supports a similar mosquito assemblage to northern Florida with many species in common, including *Cx*. *erraticus*, *Cx*. *pilosus*, and *Cx*. *quinquefasciatus* [26]. These mosquitoes are likely to co-occur with *P*. *bivittatus* in the hardwood hammocks and coniferous forests with substantial canopy cover that are preferred by pythons [77]. Mosquito populations may be substantially denser in southern Florida, which may be beneficial to *P*. *bivittatus* detection. *Aedes taeniorhynchus*, in particular, reaches high abundances at sites where *P*. *bivittatus* is common. This species was not collected in our sample from northern Florida, but may feed from *P*. *bivittatus* in the Everglades, as it is known to feed from reptiles elsewhere [78]. Unlike northern Florida, the climate of southern Florida is also conducive to year-round mosquito activity, which would be expected to benefit the use of mosquito blood meals as a source of environmental DNA for *P*. *bivittatus* detection. Additional work, particularly field study, is needed to examine mosquito-python interactions and implications in nature in the Greater Everglades Ecosystem.

Research examining the impacts of *P*. *bivittatus* on the ecology of southern Florida has focused primarily on trophic interactions between pythons and native vertebrates, particularly mammals. Beyond predation, little is known about ecological interactions between *P*. *bivittatus* and other elements of Florida’s ecosystems. In addition to the potential of using mosquitoes as a source of environmental DNA for monitoring python populations, understanding interactions between introduced *P*. *bivittatus* populations and Florida’s mosquito fauna is necessary to determine any potential role *P*. *bivittatus* may play in the transmission systems of locally-occurring parasites and pathogens. The integration of a novel, large-bodied vertebrate into Florida’s ecosystems may directly or indirectly affect the transmission dynamics of local mosquito-borne pathogens. Future research should work towards understanding these impacts, particularly on Everglades virus, as environmental change has been hypothesized to promote the epizootic emergence of other Venezuelan equine encephalitis strains [79].

## Supporting information

S1 Table Collection details and results of blood meal analysis for mosquitoes collected at the USDA National Wildlife Research Center, Gainesville, Alachua Co., Florida, USA. Each collected mosquito was given a unique identifying number (ID). The taxonomic identification (Species), date of collection (Date), sex (Sex), and the estimated extent of digestion are indicated for each mosquito specimen. A small sample of unfed female and male mosquitoes were collected to serve as negative controls to monitor for contamination and co-amplification of non-host templates. For each specimen, amplification success (Amp) and host identity (Host) are indicated.
(DOCX)
