# Supplementary material for: Interactions between the invasive Burmese python, Python bivittatus Kuhl, and the local mosquito community in Florida, USA
Source: PLoS One. 2018 Jan 17;13(1):e0190633. doi: 10.1371/journal.pone.0190633 (PMC5771569; doi:10.1371/journal.pone.0190633)
Supplement: S1 Table — Each collected mosquito was given a unique identifying number (ID). The taxonomic identification (Species), date of collection (Date), sex (Sex), and the estimated extent of digestion are indicated for each mosquito specimen. A small sample of unfed female and male mosquitoes were collected to serve as negative controls to monitor for contamination and co-amplification of non-host templates. For each specimen, amplification success (Amp) and host identity (Host) are indicated. (DOCX) [file pone.0190633.s001.docx]

**S1 Table.** Collection details and blood meal analysis results.

| **ID** | **Species** | **Date** | **Sex** | **Digestion** | **Amp** | **Host** |
| --- | --- | --- | --- | --- | --- | --- |
| PYBI01 | *Cx. erraticus* | 7/10/15 | ♀ | BF2 | + | *Python bivittatus* |
| PYBI02 | *Cx. erraticus* | 7/10/15 | ♀ | BF1 | + | *Python bivittatus* |
| PYBI03 | *Cx. erraticus* | 7/10/15 | ♀ | BF1 | + | *Python bivittatus* |
| PYBI04 | *Cx. erraticus* | 7/13/15 | ♀ | BF1 | + | *Python bivittatus* |
| PYBI05 | *Cx. erraticus* | 7/13/15 | ♂ | - | - |  |
| PYBI06 | *Cx. erraticus* | 7/13/15 | ♀ | - | - |  |
| PYBI07 | *Cx. erraticus* | 7/14/15 | ♀ | BF1 | + | *Python bivittatus* |
| PYBI08 | *Cx. erraticus* | 7/17/15 | ♀ | BF1 | + | *Python bivittatus* |
| PYBI09 | *Cx. erraticus* | 7/17/15 | ♀ | - | - |  |
| PYBI10 | *Ae. atlanticus* | 9/11/15 | ♀ | - | - |  |
| PYBI11 | *Cx. erraticus* | 9/11/15 | ♀ | BF3 | + | *Python bivittatus* |
| PYBI12 | *Cx. erraticus* | 9/16/15 | ♀ | BF1 | + | *Python bivittatus* |
| PYBI13 | *Cx. erraticus* | 9/16/15 | ♀ | BF1 | + | *Python bivittatus* |
| PYBI14 | *Ae. albopictus* | 9/16/15 | ♂ | - | - |  |
| PYBI15 | *Cx. erraticus* | 9/16/15 | ♀ | - | - |  |
| PYBI16 | *Cx. quinquefasciatus* | 9/16/15 | ♀ | - | - |  |
| PYBI17 | *An. crucians* | 9/16/15 | ♀ | - | - |  |
| PYBI18 | *Cx. erraticus* | 9/16/15 | ♀ | - | - |  |
| PYBI19 | *Cx. erraticus* | 9/16/15 | ♀ | - | - |  |
| PYBI20 | *Cx. erraticus* | 9/16/15 | ♀ | - | - |  |
| PYBI21 | *Cx. erraticus* | 9/18/15 | ♀ | BF3 | - |  |
| PYBI22 | *Cx. erraticus* | 9/18/15 | ♀ | BF1 | + | *A. carolinensis* |
| PYBI23 | *Cx. erraticus* | 9/18/15 | ♀ | - | - |  |
| PYBI24 | *Cx. pilosus* | 9/21/15 | ♀ | BF1 | + | *A. carolinensis* |
| PYBI25 | *Cx. erraticus* | 9/21/15 | ♀ | BF1 | + | *Python bivittatus* |
| PYBI26 | *Cx. erraticus* | 9/21/15 | ♀ | BF1 | + | *Python bivittatus* |
| PYBI27 | *Cx. erraticus* | 9/21/15 | ♀ | BF1 | + | *Python bivittatus* |
| PYBI28 | *Cx. erraticus* | 9/21/15 | ♀ | BF2 | + | *Python bivittatus* |
| PYBI29 | *Cx. erraticus* | 9/21/15 | ♀ | BF3 | - |  |
| PYBI30 | *Ae. albopictus* | 9/21/15 | ♀ | - | - |  |
| PYBI31 | *Cx. erraticus* | 9/22/15 | ♀ | BF1 | + | *Python bivittatus* |
| PYBI32 | *Cx. erraticus* | 9/22/15 | ♀ | BF1 | + | *Python bivittatus* |
| PYBI33 | *Cx. erraticus* | 9/22/15 | ♀ | BF1 | + | *Python bivittatus* |
| PYBI34 | *Cx. erraticus* | 9/22/15 | ♂ | - | - |  |
| PYBI35 | *Cx. erraticus* | 9/22/15 | ♀ | - | - |  |
| PYBI36 | *Cx. erraticus* | 9/22/15 | ♀ | - | - |  |
| PYBI37 | *Cx. quinquefasciatus* | 9/25/15 | ♀ | BF1 | + | *Python bivittatus* |
| PYBI38 | *Cx. erraticus* | 9/25/15 | ♀ | BF1 | + | *Python bivittatus* |
| PYBI39 | *Cx. erraticus* | 9/28/15 | ♀ | BF1 | + | *Python bivittatus* |
| PYBI40 | *Cx. erraticus* | 9/28/15 | ♀ | BF1 | + | *C. constrictor* |
| PYBI41 | *Ae. albopictus* | 9/28/15 | ♀ | - | - |  |
| PYBI42 | *Cx. quinquefasciatus* | 9/28/15 | ♀ | - | - |  |
| PYBI43 | *Cx. erraticus* | 9/29/15 | ♀ | BF1 | + | *Python bivittatus* |
| PYBI44 | *Cx. quinquefasciatus* | 9/29/15 | ♀ | - | - |  |
| PYBI45 | *Cx. pilosus* | 10/2/15 | ♀ | BF1 | + | *Python bivittatus* |
| PYBI46 | *Cx. quinquefasciatus* | 10/2/15 | ♀ | BF1 | + | *Python bivittatus* |
| PYBI47 | *Ur. lowii* | 10/21/15 | ♀ | - | - |  |
| PYBI48 | *Cx. erraticus* | 10/21/15 | ♀ | - | - |  |
| PYBI49 | *Cx. erraticus* | 10/21/15 | ♀ | - | - |  |
| PYBI50 | *Cx. erraticus* | 10/21/15 | ♀ | - | - |  |
| PYBI51 | *Cx. erraticus* | 10/27/15 | ♀ | BF3 | - |  |
| PYBI52 | *Cx. erraticus* | 5/13/16 | ♀ | BF1 | + | *F. catus* |
| PYBI53 | *Cx. erraticus* | 5/13/16 | ♀ | BF2 | + | *H. sapiens* |
| PYBI54 | *Cx. erraticus* | 5/13/16 | ♀ | BF2 | + | *Cathartes aura* |
| PYBI55 | *Cx. pilosus* | 5/25/16 | ♀ | BF1 | + | *Cathartes aura* |
| PYBI56 | *Cx. quinquefasciatus* | 6/3/16 | ♀ | BF1 | + | *Toxostoma rufum* |
| PYBI57 | *Cx. erraticus* | 7/6/16 | ♀ | BF1 | + | *Python bivittatus* |
| PYBI58 | *Cx. erraticus* | 7/6/16 | ♀ | BF1 | + | *Python bivittatus* |
| PYBI59 | *Cx. erraticus* | 7/20/16 | ♀ | BF1 | + | *Python bivittatus* |
| PYBI60 | *Cx. erraticus* | 7/20/16 | ♀ | BF1 | + | *Python bivittatus* |
| PYBI61 | *Cx. erraticus* | 7/22/16 | ♀ | BF1 | + | *Python bivittatus* |
| PYBI62 | *Cx. erraticus* | 7/22/16 | ♀ | BF2 | + | *Python bivittatus* |
| PYBI63 | *Cx. erraticus* | 7/22/16 | ♀ | BF1 | + | *Python bivittatus* |
| PYBI64 | *Cx. erraticus* | 7/25/16 | ♀ | BF1 | + | *Python bivittatus* |
| PYBI65 | *Cx. erraticus* | 7/25/16 | ♀ | BF1 | + | *Python bivittatus* |
| PYBI66 | *Cx. erraticus* | 7/27/16 | ♀ | BF1 | + | *Python bivittatus* |
| PYBI67 | *Cx. erraticus* | 7/27/16 | ♀ | BF1 | + | *Didelphis virginiana* |
| PYBI68 | *Cx. erraticus* | 8/12/16 | ♀ | BF1 | + | *Python bivittatus* |
| PYBI69 | *Cx. pilosus* | 8/12/16 | ♀ | BF1 | + | *Python bivittatus* |
| PYBI70 | *Cx. erraticus* | 8/17/16 | ♀ | BF1 | + | *Python bivittatus* |
| PYBI71 | *Cx. erraticus* | 8/17/16 | ♀ | BF1 | + | *Python bivittatus* |
| PYBI72 | *Cx. erraticus* | 8/19/16 | ♀ | BF1 | + | *Python bivittatus* |
| PYBI73 | *Cx. erraticus* | 8/19/16 | ♀ | BF2 | + | *Python bivittatus* |
| PYBI74 | *Cx. erraticus* | 8/22/16 | ♀ | BF2 | + | *Salvator merianae* |
| PYBI75 | *Cx. erraticus* | 8/26/16 | ♀ | BF1 | + | *Didelphis virginiana* |
| PYBI76 | *Cx. erraticus* | 8/29/16 | ♀ | BF1 | + | *Python bivittatus* |
| PYBI77 | *Cx. erraticus* | 8/31/16 | ♀ | BF1 | + | *Python bivittatus* |
| PYBI78 | *Cx. erraticus* | 8/31/16 | ♀ | BF1 | + | *Python bivittatus* |
| PYBI79 | *Cx. erraticus* | 8/31/16 | ♀ | BF1 | + | *Python bivittatus* |
| PYBI80 | *Cx. erraticus* | 8/31/16 | ♀ | BF1 | + | *Python bivittatus* |
| PYBI81 | *Cx. pilosus* | 9/22/16 | ♀ | BF1 | + | *Anolis carolinensis* |
| PYBI82 | *An. crucians* | 9/22/16 | ♀ | BF1 | + | *Sylvilagus floridanus* |
| PYBI83 | *An. crucians* | 9/28/16 | ♀ | BF3 | - |  |
| PYBI84 | *Cx. erraticus* | 9/28/16 | ♀ | BF2 | + | *Python bivittatus* |
| PYBI85 | *Cx. erraticus* | 9/28/16 | ♀ | BF3 | - |  |
